# Awake Fiberoptic Tracheal Intubation in a Patient With Traumatic Retropharyngeal Hematoma

**DOI:** 10.7759/cureus.48813

**Published:** 2023-11-14

**Authors:** Keigo Kani, Satoshi Yoshimura, Nobuhiro Ikeda, Nobuhiro Miyamae, Yasuyuki Sumida

**Affiliations:** 1 Emergency Medicine, Rakuwakai Otowa Hospital, Kyoto, JPN; 2 Preventive Services, Kyoto University, Kyoto, JPN

**Keywords:** emergency medicine and trauma, critical airway, cicv, neck trauma, retropharyngeal hematoma

## Abstract

Retropharyngeal hematoma is a rare disease triggered by neck trauma and can result in airway obstruction, requiring early recognition and consideration of tracheal intubation. We present a case of a 42-year-old woman brought to the emergency department with dyspnea after a traffic trauma, and a mild stridor was heard on cervical auscultation, indicating airway compromise. Contrast-enhanced computed tomography (CT) scan showed retropharyngeal hematoma. Considering her obesity and short neck, we performed awake fiberoptic intubation successfully without any complications. Awake fiberoptic intubation, directly confirming anatomic abnormalities, may increase the success rate of intubation and prevent complications, especially in patients at high risk for cannot intubate, cannot ventilate (CICV). Cervical auscultation may contribute to early diagnosis and treatment for airway obstruction in patients with cervical trauma. We report a case of awake fiberoptic tracheal intubation for a retropharyngeal hematoma in a patient at high risk for CICV and cervical auscultation in a primary survey.

## Introduction

Retropharyngeal hematoma is a rare disease in which a hematoma forms in the retropharyngeal space following trauma to the cervical region. Since hematoma formation can cause airway obstruction, rapid diagnosis is required. Although definitive airway management should be considered [1], there is no consensus on the method of airway management; oral intubation with or without awake fiberoptic intubation is the standard [2]. Here we report a case involving traumatic retropharyngeal hematoma, which was quickly diagnosed through neck auscultation during primary survey in the emergency department and successfully managed with awake fiberoptic tracheal intubation. This case shows the importance of neck auscultation and the potential of awake fiberoptic intubation to safely and successfully manage neck trauma.

## Case presentation

**Figure 1:**
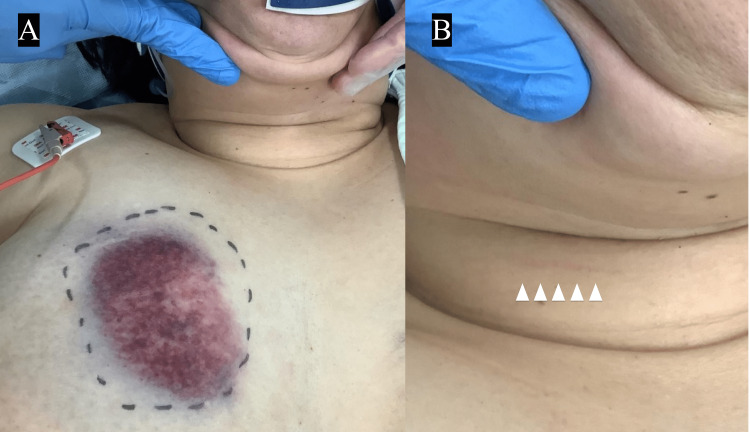
A: A contusion with hematoma in the right thoracic region (white arrow). B: A contusion in the right side of the frontal neck (white arrow).

A healthy 40-year-old woman was admitted to our emergency department with complaints of right anterior chest pain and dyspnea after a self-inflicted bicycle fall caused by brake failure. The patient hit her right anterior chest against the handlebar and a wall. The patient had a short neck and was obese, with a body mass index (BMI) of 32. On the primary survey, airway obstruction was not identifiable and the SpO2 was 100% on ambient air, however, the patients had tachypnea with a respiratory rate of 24 breaths/min. In terms of the hemodynamic system, the patients had tachycardia of pule rate with 130/min, and with a blood pressure of 146/121mmHg. A focused assessment with sonography for trauma (FAST) was performed immediately after hospital arrival and was negative. There were no abnormal findings on chest and pelvic x-rays. The patient was alert and we moved to the secondary survey. Tenderness in the anterior neck and a hematoma in the right chest wall was revealed (Figures 1A, 1B). In addition to the usual secondary survey, we successively performed the auscultation of the neck because of the continued dyspnea of the patient, which identified stridor on the neck - the indicative sign of airway obstruction. 

**Figure 2:**
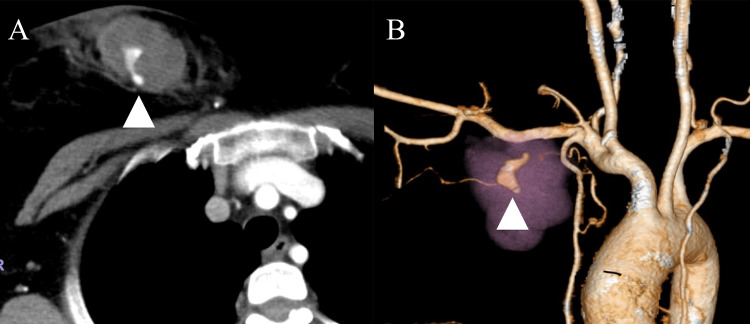
Computed tomography revealed a right anterior thoracic hematoma with extravasation (A, horizontal axial view (white arrow heads), and B, three-dimension construction view (white arrow heads))

**Figure 3:**
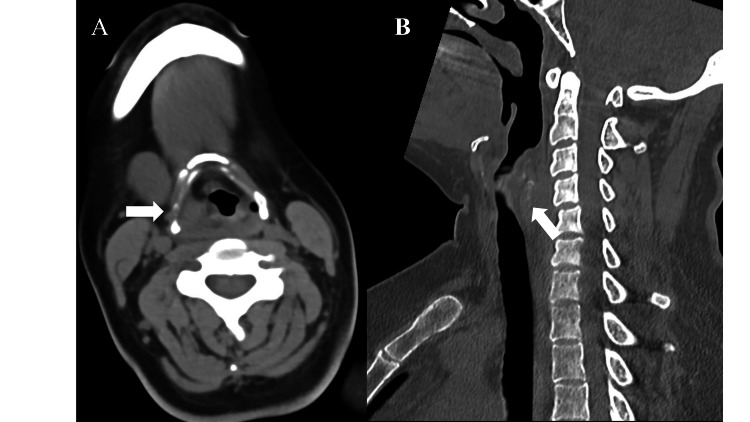
A retropharyngeal hematoma without extravasation (A, horizontal axis view (white arrow), and B, sagittal axis view (white arrow)).

The head, cervical, chest, and abdominal contrast-enhanced CT were performed. to evaluate trauma, revealing a right anterior chest wall hematoma with extravasation (Figures 2A, 2B) and a retropharyngeal hematoma without extravasation (Figures 3A, 3B). After CT, the patient still complained of dyspnea and post-traumatic sore throat even under treatment with oxygen therapy with a non-rebreather mask. Upper airway obstruction due to the retropharyngeal hematoma was suspected based on the physical examination and CT findings. There was no evidence of other airway obstruction due to blunt neck trauma, such as a crushed larynx or a tracheal disruption.

**Figure 4:**
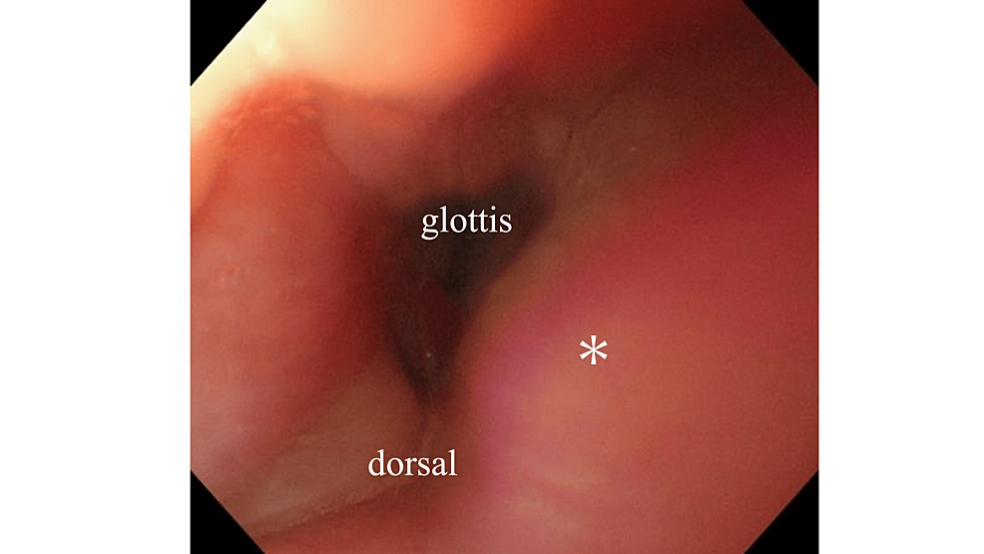
Bronchoscopy revealed mucosal edema due to retropharyngeal hematoma on the right dorsal side (white asterisk).

Tracheal intubation was decided on for the management of the airway obstruction. To mitigate the risk of iatrogenic injury to the airway, fiberoptic intubation was chosen for airway management. This choice was made due to the superior visualization of the glottis and retropharyngeal hematoma offered by fiberoptic intubation when compared to the video laryngoscope. We decided to perform awake fiberoptic intubation because the patient was considered at high risk for cannot intubate, cannot ventilate (CICV) due to her obesity, short neck, and abnormal airway anatomy derived from the hematoma. We had emergency physicians trained for both surgical and non-surgical airway management at the scene and a surgical airway management kit was prepared in the department for the backup airway plan. A 7.5 mm tube was inserted orally under bronchoscopy with intravenous administration of fentanyl 100 μg and midazolam 5 mg. We have chosen the tube size according to the sex of the patient and the size of the bronchoscopy. During intubation, bronchoscopy revealed mucosal edema due to retropharyngeal hematoma on the right dorsal side (Figure 4). The patient was intubated without complications and admitted to the intensive care unit. The right anterior chest wall hematoma was treated by compression hemostasis using a sandbag without any endovascular therapy. 

On day two, a laryngoscopy confirmed improvement of the mucosal edema, and the patient was extubated. She was moved from the intensive care unit (ICU) to the general ward on day three due to her stabilized condition and ability to take oral medication. The patient was discharged from the hospital on day four without any airway complications. The right anterior chest wall hematoma was hemostatic on day two confirmed by bedside ultrasound, which showed no inflow of blood, and sandbag compression was completed.

## Discussion

In the present case, the patient with traumatic retropharyngeal hematoma was successfully managed with awake fiberoptic intubation at an early admission stage and could be discharged from the hospital without any complication. Retropharyngeal hematoma is often triggered by blunt trauma to the neck and can progress to airway obstruction [1]. The mortality rate of retropharyngeal hematoma is 18.2%, and the leading cause of death is airway obstruction and concomitant cervical spine injury [3]. Because hematoma formation leads to airway obstruction, tracheal intubation should be considered [1,4]. In previous reports, some patients did not require intubation [3,5]. Still, others required intubation five to 20 hours after admission, often when patients did not need intubation at presentation to the emergency department [6,7]. We have decided the intubation for the patient to avoid the developing hypo-oxygenation because the patient was considered to be at high risk of respiratory failure because of airway obstruction on the cervical CT scan and persistent and worsened tachypnea and dyspnea regardless of maximum O2 supply via a mask.

In this case, the critical points are that neck auscultation is a clue for the early diagnosis of retropharyngeal hematoma, and that awake fiberoptic intubation is recommended because the patient is at risk for CICV. In the current case, the cause of respiratory distress was unknown just after arrival at the hospital, as we could not identify a low SpO2 or a foreign body in the oral cavity, although the patient was tachypneic. The Advanced Trauma Life Support® (ATLS), European Trauma Course (ETC) guidelines [8,9] do not recommend auscultation of the neck in the primary survey. However, in cases such as the present case, when neck trauma is suspected from the origin of the injury, it is vital to evaluate stridor by auscultation of the neck during the primary survey at the emergency department, as this may allow early diagnosis and treatment of the cause of the compromised airway.

Second, although securing the airway is known to be essential for this condition [1], there has yet to be a consensus on the optimal method of airway management, with techniques ranging from oral intubation to awake fiberoptic intubation have been reported [2]. There may be a considerable disadvantage in choosing oral intubation for airway management in patients with retropharyngeal hematomas. Several previous studies have reported that 13.5% of oral intubations for retropharyngeal hematoma failed [3] and that the risk of airway compromise due to bleeding and exacerbation of edema caused by rupture of the hematoma was associated with the intubation procedure [1,6]. In addition, in a prior case report of a patient with a short neck and obesity, as in the present case, and with an anatomic structural abnormality of the airway due to hematoma, the administration of drugs during intubation increased the risk of CICV [10]. In contrast, awake fiberoptic intubation prevents airway collapse by drug administration, has been shown to preserve spontaneous breathing, and may secure the airway without exacerbating airway compromise by directly confirming the anatomic abnormalities of the airway [1,6,11].

## Conclusions

This case highlights that cervical auscultation in the primary survey can identify airway compromise in the early stages. Awake fiberoptic intubation improves the prognosis of patients with retropharyngeal hematoma in those at high risk for CICV. Retropharyngeal hematoma is associated with anatomic abnormalities of the airway, and in addition, when the risk of CICV is high, inadequate intubation technique can worsen the patient's prognosis. Early diagnosis of airway compromise by cervical auscultation can contribute to estimating the risk of difficulties in intubation and determining the appropriate technique.
